# Erratum for Ma, “A new hypothesis on BV etiology: dichotomous and crisscrossing categorization of complex versus simple on healthy versus BV vaginal microbiomes”

**DOI:** 10.1128/msystems.00687-24

**Published:** 2024-07-23

**Authors:** Zhanshan (Sam) Ma

## ERRATUM

Vol. 8, no. 5, 2023, e00049-23, https://doi.org/10.1128/msystems.00049-23. Page 24: At the time of publication, reference 40 was under review at a scholarly journal and should therefore appear as “Ma ZS. Submitted for publication.”

