# Supplementary material for: A new hypothesis on BV etiology: dichotomous and crisscrossing categorization of complex versus simple on healthy versus BV vaginal microbiomes
Source: mSystems. 2023 Aug 30;8(5):e00049-23. doi: 10.1128/msystems.00049-23 (PMC10654060; doi:10.1128/msystems.00049-23)
Supplement: Supplemental Tables — Tables S1-S6, S9, and S10. [file msystems.00049-23-s0002.pdf]

Online Supplementary Information for: Ma ZS (2022) A new hypothesis on BV etiology: dichotomous and crisscrossing categorization of complex vs. simple upon healthy vs. BV vaginal microbiomes

List of Online Supplementary Tables S1-S10

Table S1. Brief information on the BV microbiome datasets

Table S2. The *mean* and *standard error* of the Hill numbers (diversity) and community dominance across four groups for regrouped BV microbiome datasets, as well as the results of Wilcoxon tests

Table S3. The results of shared species analysis between four VM types

Table S4A. Fitting to Sloan's (2006) near neutral model with VM datasets
Table S4B. The *P*-value of Fisher's test for the percentage of below-neutral, neutral and above-neutral species from Sloan modeling with both source and destination community are the same

Table S5A. The mean of the similarity (S) and normalized stochasticity ratio (*NSR*)
Table S5B. The *P*-values of the Wilcoxon tests for the similarity (S) and normalized stochasticity ratio (*NSR*)

Table S6A. The TPLE (Taylor's power law extension) parameters for the four VM types
Table S6B. The results of permutation tests for the TPLE parameters of the four VM types

Table S7 & Table S8 are stored in MS-Excel Sheets
Table S7A. The species specificity (SS) of **Healthy-C and Healthy-S** microbiome
Table S7B. The species specificity (SS) of **BV-C and BV-S** microbiome
Table S7C. The species specificity (SS) of **Healthy-S and BV-S** microbiome
Table S7D. The species specificity (SS) of **Healthy-C and BV-C** microbiome

Table S8A. The lists of species groups (SG) with significant differences in species specificity (SS) between **Healthy-C and Healthy-S** microbiome
Table S8B. The lists of species groups (SG) with significant differences in species specificity (SS) between **BV-C and BV-S** microbiome
Table S8C. The lists of species groups (SG) with significant differences in species specificity (SS) between **Healthy-S and BV-S** microbiome
Table S8D. The lists of species groups (SG) with significant differences in species specificity (SS) between **Healthy-C and BV-C** microbiome
Table S8E. The lists of species groups (SG) with significant differences in species specificity (SS) between **Healthy-C and BV-S** microbiome
Table S8F. The lists of species groups (SG) with significant differences in species specificity (SS) between **Healthy-S and BV-C** microbiome

Table S9A. Permutation tests for the SD (specificity diversity) of the species group (SG) of "Unique Species in the former (healthy or complex community)" with 1000 times of re-sampling
Table S9B. Permutation tests for the SD (specificity diversity) of the species group (SG) of "Unique Species in the latter (BV or simple community)" with 1000 times of re-sampling
Table S9C. Permutation tests for the SD (specificity diversity) of the species group (SG) of "Enriched Species in the former (healthy or complex) community" with 1000 times of re-sampling
Table S9D. Permutation tests for the SD (specificity diversity) of the species group (SG) of "Enriched Species in the latter (BV or simple community)" with 1000 times of re-sampling
Table S9E. Permutation tests for the SD (specificity diversity) of the species group (SG) of "All species with significant differences in specificity" with 1000 times of re-sampling
Table S9F. Permutation tests for the SD (specificity diversity) of the species group (SG) of "All species" with 1000 times of re-sampling

Table S10. The mean accuracy level from 100 repeated learning operations with randomly split of 75% samples for training (learning) and 25% samples for model evaluations (testing)

Table S1. Brief information on the BV microbiome datasets

Dataset No.	NCBI Data Accession No.	Group	Sample Size	Reference
Dataset #1	ERP022287	Healthy	20	Gottschick C, et al. (2017) *Microbiome*
		BV	236	
Dataset #2	ERP113320	Healthy	215	Koirala R, et al. (2020) *FEMS Microbiol Ecol*
Dataset #3	SRP002463	Healthy	981	Gajer P, Brotman RM, Bai G, et al. (2012) *Sci Transl Med*
Dataset #4	SRP026107	Healthy	328	Ravel J, Bortman RM, Gajer P, et al. (2013) *Microbiome*
		BV	1657	
Dataset #5	SRP056030	Healthy	20	Srinivasan S, et al. (2015) *mBio.*
		BV	40	
Dataset #6	SRP066130	BV	68	Xiao B, et al. (2016) *Sci. Rep.*
Dataset #7	SRP124970	Healthy	166	Muzny CA, et al. (2018) *J Infect Dis*
		BV	277	
Dataset #8	SRP174636	Healthy	161	Wang C, et al. (2020) *Diagn Microbiol Infect Dis*
Dataset #9	SRP186938	Healthy	37	Zwittink RD, et al. (2020) *Eur J Clin Microbiol Infect Dis.*
		BV	49	
Dataset #10	SRP187026	Healthy	21	Ceccarani C, et al. (2019) *Sci Rep*
		BV	20	
Dataset #11	SRP201810	BV	1270	Tettamanti Boshier FA, et al. (2020) *mSystems*
Dataset #12	SRP218617	Healthy	98	Tortelli BA, et al. (2020) *Am J Obstet Gynecol*
Dataset #14	SRP266775	Healthy	117	Ahrens P, Andersen LO, Lilje B, et al. (2020) *PLoS One*
Total sample size of BV microbiome			**3617**	
Total sample size of Healthy microbiome			**3265**	

Table S2. The *mean* and *standard error* of the Hill numbers (diversity) and community dominance across four groups for regrouped BV microbiome datasets, as well as the results of Wilcoxon tests

**Group**	**Statistics**	***q* = 0**	***q* = 1**	***q* = 2**	***q* = 3**	**Community Dominance**
Healthy-C	Mean	28.534	8.098	5.548	4.749	126.898
	Std. Err.	0.561	0.127	0.083	0.068	1.472
Healthy-S	Mean	13.440	2.314	1.901	1.818	314.748
	Std. Err.	0.316	0.017	0.012	0.011	2.440
Healthy-C vs. Healthy-S	≠	0.000	0.000	0.000	0.000	0.000
	<	1.000	1.000	1.000	1.000	0.000
	>	0.000	0.000	0.000	0.000	1.000
BV-C	Mean	29.941	7.819	5.270	4.508	136.065
	Std. Err.	0.591	0.113	0.072	0.060	1.932
BV-S	Mean	14.422	2.206	1.611	1.488	364.794
	Std. Err.	0.216	0.017	0.010	0.008	2.161
BV-C vs. BV-S	≠	0.000	0.000	0.000	0.000	0.000
	<	1.000	1.000	1.000	1.000	0.000
	>	0.000	0.000	0.000	0.000	1.000
**Group**	**Statistics**	***q* = 0**	***q* = 1**	***q* = 2**	***q* = 3**	**Community Dominance**
Healthy-C vs. BV-C	≠	0.018	0.811	0.041	0.007	0.062
	<	0.009	0.595	0.979	0.996	0.031
	>	0.991	0.405	0.021	0.004	0.969
Healthy-S vs. BV-S	≠	0.000	0.000	0.000	0.000	0.000
	<	0.000	1.000	1.000	1.000	0.000
	>	1.000	0.000	0.000	0.000	1.000
Healthy-C *vs*. BV-S	≠	0.000	0.000	0.000	0.000	0.000
	<	1.000	1.000	1.000	1.000	0.000
	>	0.000	0.000	0.000	0.000	1.000
Healthy-S vs. BV-C	≠	0.000	0.000	0.000	0.000	0.000
	<	0.000	0.000	0.000	0.000	1.000
	>	1.000	1.000	1.000	1.000	0.000

Table S3. The results of shared species analysis between four VM types

**Group**	**Observed Shared OTUs**	**Reads Randomization**		**Samples Randomization**	
		**Expected Shared OTUs**	***P*-value**	**Expected Shared OTUs**	***P*-value**
Healthy-S vs. Healthy-C	384	549.450	0.000	426.965	0.000
BV-S vs. BV-C	282	495.978	0.000	406.160	0.000
Healthy-C vs. BV-C	404	601.912	0.000	475.464	0.000
Healthy-S vs. BV-S	262	495.978	0.000	324.412	0.000
Healthy-C vs. BV-S	272	517.498	0.000	410.812	0.000
Healthy-S vs. BV-C	363	610.650	0.000	442.609	0.000

Table S4A. Fitting to Sloan's (2006) near neutral model with VM datasets

Source Community	Destination Community	*N* (Average Reads)	Immigration Probability (*m*)	R^2	Total Number of Species	Percentage of Species Below Neutral (%)	Percentage of Neutral Species (%)	Percentage of Species Above Neutral (%)
Healthy-C	Healthy-C	27645	0.000	0.490	483	9.3	37.5	53.2
Healthy-S	Healthy-S	30194	0.000	0.708	451	5.5	56.1	38.4
BV-C	BV-C	43772	0.000	0.610	523	6.1	40.3	53.5
BV-S	BV-S	24435	0.001	0.665	307	10.4	49.8	39.7
Healthy-C	Healthy-S	30194	0.000	0.579	384	14.6	47.7	37.8
Healthy-S	Healthy-C	27645	0.002	0.254	384	14.1	30.2	55.7
BV-C	BV-S	24435	0.000	0.494	282	12.1	38.7	49.3
BV-S	BV-C	43772	0.003	0.511	282	17.4	19.5	63.1
Healthy-C	BV-C	43772	0.000	0.376	404	15.6	30.0	54.5
BV-C	Healthy-C	24435	0.000	0.579	262	18.3	34.4	47.3
Healthy-S	BV-S	27645	0.000	0.320	404	5.9	25.0	69.1
BV-S	Healthy-S	30194	0.000	0.665	262	4.6	28.2	67.2
**Mean**		31512	0.001	0.521	369	11.2	36.5	52.4
**Standard Error**		2056	0.000	0.038	24	1.3	2.9	2.8

Table S4B. The *P*-value of Fisher's test for the percentage of below-neutral, neutral and above-neutral species from Sloan modeling with both source and destination community are the same

Comparisons	Percentage of Species Below Neutral (%)	Percentage of Neutral Species (%)	Percentage of Species Above Neutral (%)
Healthy-C *vs*. Healthy-S	0.047	0.001	0.006
BV-C *vs*. BV-S	0.045	0.106	0.022
Healthy-C *vs*. BV-C	0.097	0.551	0.957
Healthy-S *vs*. BV-S	0.025	0.379	0.834

Table S5A. The mean of the similarity (S) and normalized stochasticity ratio (*NSR*)

Group	*Number of Comparisons*	Similarity (*S*)	Normalized Stochasticity Ratio (*NSR*)
Healthy-C	1194285	0.834	0.354
Healthy-S	1476621	0.794	0.403
BV-C	1322751	0.836	0.376
BV-S	1979055	0.743	0.326

Table S5B. The *P*-values of the Wilcoxon tests for the similarity (S) and normalized stochasticity ratio (*NSR*)

Group	Similarity (*S*)			Normalized Stochasticity Ratio (*NSR*)		
	≠	>	<	≠	>	<
Healthy-C *vs*. Healthy-S	0.000	0.000	1.000	0.000	1.000	0.000
BV-C *vs*. BV-S	0.000	0.000	1.000	0.000	0.000	1.000
Healthy-C *vs*. BV-C	0.000	0.000	1.000	0.000	0.000	1.000
Healthy-S *vs*. BV-S	0.000	0.000	1.000	0.000	0.000	1.000
Healthy-C vs. BV-S	0.000	0.000	1.000	0.000	0.000	1.000
Healthy-S vs. BV-C	0.000	1.000	0.000	0.000	0.000	1.000

Table S6A. The TPLE (Taylor's power law extension) parameters for the four VM types

Group	*b*	ln(*a*)	CACD	*R*	*p*–value	*n*
Healthy-C	1.971	4.683	0.008	0.987	0.000	1546
Healthy-S	2.033	5.390	0.005	0.994	0.000	1719
BV-C	1.874	5.161	0.003	0.983	0.000	1627
BV-S	2.017	5.213	0.006	0.991	0.000	1990

Table S6B. The results of permutation tests for the TPLE parameters of the four VM types

Group	*b*	ln(*a*)	CACD
Healthy-S vs. Healthy-C	0.000	0.000	0.008
BV-C vs. BV-C	0.000	0.660	0.000
Healthy-C vs. BV-C	0.000	0.000	0.000
Healthy-S vs. BV-S	0.042	0.095	0.422
Healthy-S vs. BV-C	0.000	0.006	0.000
Healthy-C vs. BV-S	0.007	0.000	0.103
Percentage (%) with significant differences	**100%**	**67%**	**67%**

Table S9A. Permutation tests for the SD (specificity diversity) of the species group (SG) of “Unique Species in the former (healthy or complex community)” with 1000 times of re-sampling

Comparison	Order	Former	Latter	Observed= Former-Latter	Lower	Upper	*p*-value
Healthy-C vs. Healthy-S	*q*=0	99.000	0.000	99.000	-27.429	18.035	0.000
	q=1	40.694	0.000	40.694	-21.980	18.908	0.000
	q=2	24.420	0.000	24.420	-16.242	14.890	0.002
	q=3	19.561	0.000	19.561	-12.942	12.001	0.002
	q=4	17.375	0.000	17.375	-11.188	10.390	0.002
BV-C vs. BV-S	*q*=0	241.000	0.000	241.000	-78.625	46.781	0.000
	q=1	131.319	0.000	131.319	-55.980	41.878	0.000
	q=2	84.819	0.000	84.819	-46.784	40.137	0.000
	q=3	63.646	0.000	63.646	-42.810	38.570	0.000
	q=4	52.211	0.000	52.211	-39.462	36.059	0.000
Healthy-S vs. BV-S	*q*=0	189.000	0.000	189.000	-73.488	50.850	0.000
	q=1	99.499	0.000	99.499	-65.042	53.798	0.000
	q=2	42.386	0.000	42.386	-61.497	58.251	0.186
	q=3	23.038	0.000	23.038	-52.068	51.409	0.440
	q=4	16.798	0.000	16.798	-43.071	42.980	0.490
Healthy-C vs. BV-C	*q*=0	79.000	0.000	79.000	-25.727	21.503	0.000
	q=1	48.901	0.000	48.901	-26.750	24.306	0.000
	q=2	29.434	0.000	29.434	-27.057	25.486	0.024
	q=3	20.136	0.000	20.136	-25.567	24.327	0.107
	q=4	15.904	0.000	15.904	-23.581	22.481	0.186
Healthy-C vs. BV-S	*q*=0	211.000	0.000	211.000	-48.941	18.561	0.000
	q=1	82.469	0.000	82.469	-37.491	28.393	0.000
	q=2	49.136	0.000	49.136	-23.398	19.464	0.000
	q=3	38.010	0.000	38.010	-18.233	15.696	0.000
	q=4	32.486	0.000	32.486	-16.511	14.547	0.000
Healthy-S vs. BV-C	*q*=0	7.000	0.000	7.000	-0.698	0.740	0.000
	q=1	4.225	0.000	4.225	-3.641	3.762	0.014
	q=2	2.808	0.000	2.808	-3.603	3.695	0.126
	q=3	2.319	0.000	2.319	-3.195	3.268	0.155
	q=4	2.125	0.000	2.125	-2.894	2.957	0.142
Percentage (%) with significant differences	*q*=0						100
	q=1						100
	q=2						66.7
	q=3						50
	q=4						50

Table S9B. Permutation tests for the SD (specificity diversity) of the species group (SG) of “Unique Species in the latter (BV or simple community)” with 1000 times of re-sampling

Comparison	Order	Former	Latter	Observed= Former-Latter	Lower	Upper	*p*-value
Healthy-C vs. Healthy-S	*q*=0	0.000	2.000	-2.000	-0.703	0.617	0.000
	q=1	0.000	1.890	-1.890	-1.192	1.113	0.000
	q=2	0.000	1.800	-1.800	-1.289	1.203	0.000
	q=3	0.000	1.732	-1.732	-1.295	1.206	0.000
	q=4	0.000	1.683	-1.683	-1.282	1.192	0.000
BV-C vs. BV-S	*q*=0	NA	NA	NA	NA	NA	NA
	q=1	NA	NA	NA	NA	NA	NA
	q=2	NA	NA	NA	NA	NA	NA
	q=3	NA	NA	NA	NA	NA	NA
	q=4	NA	NA	NA	NA	NA	NA
Healthy-S vs. BV-S	*q*=0	0.000	6.000	-6.000	-1.147	0.963	0.000
	q=1	0.000	5.752	-5.752	-1.915	1.614	0.000
	q=2	0.000	5.531	-5.531	-2.257	1.962	0.000
	q=3	0.000	5.342	-5.342	-2.347	2.073	0.000
	q=4	0.000	5.185	-5.185	-2.359	2.103	0.000
Healthy-C vs. BV-C	*q*=0	0.000	25.000	-25.000	-2.756	2.434	0.000
	q=1	0.000	15.100	-15.100	-9.085	8.733	0.000
	q=2	0.000	8.640	-8.640	-8.422	8.125	0.032
	q=3	0.000	6.263	-6.263	-6.814	6.569	0.062
	q=4	0.000	5.323	-5.323	-5.791	5.577	0.068
Healthy-C vs. BV-S	*q*=0	0.000	5.000	-5.000	-1.158	0.870	0.000
	q=1	0.000	4.761	-4.761	-1.841	1.376	0.000
	q=2	0.000	4.556	-4.556	-2.112	1.631	0.000
	q=3	0.000	4.392	-4.392	-2.166	1.701	0.000
	q=4	0.000	4.264	-4.264	-2.161	1.714	0.000
Healthy-S vs. BV-C	*q*=0	0.000	160.000	-160.000	-44.834	51.430	0.000
	q=1	0.000	52.790	-52.790	-39.929	41.838	0.003
	q=2	0.000	18.839	-18.839	-24.366	24.720	0.143
	q=3	0.000	11.739	-11.739	-15.771	15.916	0.147
	q=4	0.000	9.380	-9.380	-12.226	12.330	0.137
Percentage (%) with significant differences	*q*=0						100
	q=1						100
	q=2						80
	q=3						60
	q=4						60

Table S9C. Permutation tests for the SD (specificity diversity) of the species group (SG) of "Enriched Species in the former (healthy or complex) community" with 1000 times of re-sampling

Comparison	Order	Former	Latter	Observed= Former-Latter	Lower	Upper	*p*-value
Healthy-C vs. Healthy-S	q=0	205.000	205.000	0.000	-0.979	0.845	0.069
	q=1	110.195	35.050	75.146	-13.691	12.474	0.000
	q=2	75.774	16.881	58.893	-10.943	10.249	0.000
	q=3	60.114	12.276	47.838	-8.652	8.188	0.000
	q=4	51.761	10.408	41.353	-7.445	7.091	0.000
BV-C vs. BV-S	q=0	182.000	182.000	0.000	-0.388	0.352	0.036
	q=1	87.971	37.878	50.093	-10.600	9.579	0.000
	q=2	60.679	19.125	41.553	-6.798	6.310	0.000
	q=3	50.755	13.080	37.675	-5.256	4.956	0.000
	q=4	45.721	10.486	35.234	-4.611	4.401	0.000
Healthy-S vs. BV-S	q=0	64.000	64.000	0.000	-0.426	0.370	0.039
	q=1	23.201	5.980	17.221	-4.522	4.289	0.000
	q=2	10.072	3.404	6.668	-1.882	1.788	0.000
	q=3	6.858	2.990	3.868	-1.238	1.175	0.000
	q=4	5.717	2.836	2.881	-1.016	0.964	0.000
Healthy-C vs. BV-C	q=0	115.000	115.000	0.000	-0.198	0.194	0.010
	q=1	69.828	24.008	45.820	-9.200	8.901	0.000
	q=2	47.897	15.209	32.688	-6.866	6.670	0.000
	q=3	36.073	12.813	23.260	-5.049	4.923	0.000
	q=4	29.387	11.711	17.676	-4.156	4.070	0.000
Healthy-C vs. BV-S	q=0	174.000	174.000	0.000	-0.871	0.677	0.135
	q=1	97.301	30.414	66.886	-9.715	9.042	0.000
	q=2	68.880	16.474	52.407	-7.157	6.970	0.000
	q=3	55.666	11.846	43.820	-5.486	5.366	0.000
	q=4	48.595	9.767	38.828	-4.827	4.706	0.000
Healthy-S vs. BV-C	q=0	23.000	23.000	0.000	-0.273	0.281	0.020
	q=1	10.937	7.056	3.881	-1.115	1.077	0.000
	q=2	8.077	5.034	3.043	-0.877	0.827	0.000
	q=3	7.021	4.203	2.817	-0.752	0.705	0.000
	q=4	6.471	3.774	2.697	-0.685	0.642	0.000
Percentage (%) with significant differences	q=0						66.7
	q=1						100
	q=2						100
	q=3						100
	q=4						100

Table S9D. Permutation tests for the SD (specificity diversity) of the species group (SG) of "Enriched Species in the latter (BV or simple community)" with 1000 times of re-sampling

Comparison	Order	Former	Latter	Observed= Former-Latter	Lower	Upper	*p*-value
Healthy-C vs. Healthy-S	*q*=0	17.000	17.000	0.000	0.000	0.000	0.000
	q=1	6.118	9.073	-2.955	-0.936	0.854	0.000
	q=2	4.852	7.004	-2.152	-0.655	0.603	0.000
	q=3	4.309	6.289	-1.980	-0.551	0.511	0.000
	q=4	3.966	5.946	-1.980	-0.508	0.473	0.000
BV-C vs. BV-S	*q*=0	10.000	10.000	0.000	0.000	0.000	0.000
	q=1	6.474	7.421	-0.946	-0.504	0.470	0.000
	q=2	5.545	6.680	-1.136	-0.531	0.502	0.000
	q=3	5.068	6.324	-1.256	-0.550	0.527	0.000
	q=4	4.783	6.111	-1.328	-0.564	0.544	0.000
Healthy-S vs. BV-S	*q*=0	55.000	55.000	0.000	-0.090	0.086	0.002
	q=1	17.385	29.282	-11.897	-2.264	2.298	0.000
	q=2	11.408	21.134	-9.726	-1.882	1.994	0.000
	q=3	9.015	17.582	-8.567	-1.747	1.878	0.000
	q=4	7.766	15.572	-7.807	-1.689	1.828	0.000
Healthy-C vs. BV-C	*q*=0	77.000	77.000	0.000	-0.108	0.106	0.003
	q=1	23.028	41.121	-18.094	-3.041	2.951	0.000
	q=2	16.651	31.575	-14.924	-2.293	2.209	0.000
	q=3	14.427	27.523	-13.096	-2.062	1.982	0.000
	q=4	13.299	25.082	-11.783	-1.950	1.876	0.000
Healthy-C vs. BV-S	*q*=0	17.000	17.000	0.000	0.000	0.000	0.000
	q=1	6.790	8.849	-2.058	-0.650	0.618	0.000
	q=2	6.262	7.146	-0.884	-0.439	0.411	0.000
	q=3	6.093	6.504	-0.411	-0.408	0.380	0.037
	q=4	5.989	6.139	-0.150	-0.413	0.384	0.482
Healthy-S vs. BV-C	*q*=0	190.000	190.000	0.000	-0.351	0.361	0.022
	q=1	43.786	82.043	-38.257	-11.802	11.991	0.000
	q=2	22.222	55.861	-33.638	-7.421	7.452	0.000
	q=3	16.346	46.808	-30.462	-5.815	5.828	0.000
	q=4	13.957	42.186	-28.228	-5.135	5.149	0.000
Percentage (%) with significant differences	*q*=0						100
	q=1						100
	q=2						100
	q=3						100
	q=4						83.3

Table S9E. Permutation tests for the SD (specificity diversity) of the species group (SG) of "All species with significant differences in specificity" with 1000 times of re-sampling

Comparison	Order	Former	Latter	Observed= Former-Latter	Lower	Upper	*p*-value
Healthy-C vs. Healthy-S	*q*=0	321.000	224.000	97.000	-27.731	18.117	0.000
	q=1	125.682	20.556	105.126	-15.116	13.622	0.000
	q=2	83.725	11.045	72.680	-10.375	9.756	0.000
	q=3	66.029	9.030	56.999	-8.052	7.690	0.000
	q=4	56.659	8.217	48.441	-6.962	6.698	0.000
BV-C vs. BV-S	*q*=0	433.000	192.000	241.000	-78.668	46.788	0.000
	q=1	111.732	24.286	87.445	-16.635	15.184	0.000
	q=2	70.350	13.902	56.448	-7.950	7.444	0.000
	q=3	57.649	11.510	46.138	-6.016	5.711	0.000
	q=4	51.429	10.500	40.929	-5.332	5.113	0.000
Healthy-S vs. BV-S	*q*=0	308.000	125.000	183.000	-73.690	50.808	0.000
	q=1	54.757	35.082	19.675	-7.889	7.521	0.000
	q=2	22.821	24.361	-1.540	-3.578	3.577	0.373
	q=3	14.941	20.190	-5.249	-2.803	2.849	0.000
	q=4	11.918	17.820	-5.902	-2.501	2.558	0.000
Healthy-C vs. BV-C	*q*=0	271.000	217.000	54.000	-26.029	21.477	0.000
	q=1	98.934	63.085	35.850	-11.031	10.514	0.000
	q=2	66.261	43.805	22.456	-7.371	7.060	0.000
	q=3	50.953	36.905	14.048	-5.714	5.477	0.000
	q=4	41.927	32.994	8.933	-4.937	4.743	0.000
Healthy-C vs. BV-S	*q*=0	402.000	196.000	206.000	-49.319	18.457	0.000
	q=1	134.404	25.036	109.368	-15.880	14.450	0.000
	q=2	86.134	14.208	71.926	-8.966	8.621	0.000
	q=3	67.298	11.489	55.809	-6.612	6.366	0.000
	q=4	57.748	10.280	47.468	-5.676	5.446	0.000
Healthy-S vs. BV-C	*q*=0	220.000	373.000	-153.000	-44.856	51.512	0.000
	q=1	24.359	97.516	-73.157	-14.304	14.595	0.000
	q=2	12.414	62.478	-50.064	-8.045	8.030	0.000
	q=3	9.831	51.526	-41.696	-6.620	6.566	0.000
	q=4	8.737	46.086	-37.349	-6.124	6.062	0.000
Percentage (%) with significant differences	*q*=0						100
	q=1						100
	q=2						83.3
	q=3						100
	q=4						100

Table S9F. Permutation tests for the SD (specificity diversity) of the species group (SG) of "All species" with 1000 times of re-sampling

Comparison	Order	Former	Latter	Observed= Former - Latter	Lower	Upper	*p*-value
Healthy-C vs. Healthy-S	*q*=0	483.000	451.000	32.000	-62.710	38.376	0.256
	q=1	142.699	34.979	107.720	-20.219	17.125	0.000
	q=2	90.861	15.595	75.266	-11.862	10.729	0.000
	q=3	70.916	12.076	58.840	-9.009	8.327	0.000
	q=4	60.539	10.713	49.826	-7.744	7.235	0.000
BV-C vs. BV-S	*q*=0	523.000	307.000	216.000	-86.502	46.188	0.000
	q=1	120.412	31.024	89.388	-19.482	17.506	0.000
	q=2	73.237	16.021	57.217	-8.804	8.167	0.000
	q=3	59.530	12.918	46.612	-6.520	6.138	0.000
	q=4	52.933	11.650	41.283	-5.717	5.441	0.000
Healthy-S vs. BV-S	*q*=0	451.000	307.000	144.000	-85.091	51.525	0.000
	q=1	79.537	56.335	23.202	-12.277	11.551	0.000
	q=2	33.706	35.257	-1.551	-5.426	5.383	0.568
	q=3	22.194	28.465	-6.271	-4.183	4.201	0.005
	q=4	17.517	24.824	-7.308	-3.679	3.712	0.000
Healthy-C vs. BV-C	*q*=0	483.000	523.000	-40.000	-60.334	48.378	0.165
	q=1	141.097	99.559	41.539	-18.895	17.694	0.000
	q=2	88.392	61.353	27.039	-10.807	10.199	0.000
	q=3	66.757	49.702	17.055	-8.138	7.694	0.000
	q=4	54.275	43.530	10.745	-6.931	6.569	0.001
Healthy-C vs. BV-S	*q*=0	483.000	307.000	176.000	-60.641	18.369	0.000
	q=1	146.537	33.718	112.819	-18.199	16.174	0.000
	q=2	91.535	17.425	74.111	-10.082	9.550	0.000
	q=3	70.844	13.603	57.241	-7.434	7.068	0.000
	q=4	60.493	11.969	48.525	-6.356	6.031	0.000
Healthy-S vs. BV-C	*q*=0	451.000	523.000	-72.000	-62.212	77.970	0.040
	q=1	37.815	110.973	-73.158	-19.796	20.716	0.000
	q=2	16.152	67.207	-51.055	-9.865	9.978	0.000
	q=3	12.262	54.676	-42.415	-7.696	7.707	0.000
	q=4	10.690	48.624	-37.933	-6.945	6.923	0.000
Percentage (%) with significant differences	*q*=0						66.7
	q=1						100
	q=2						83.3
	q=3						100
	q=4						100

Table S10. The mean accuracy level from 100 repeated learning operations with randomly split of 75% samples for training (learning) and 25% samples for model evaluations (testing)

Input Data	Comparisons	Neuron Network	Random Forest	Support Vector Machine	Logistic Regression	Gradient Boosting	K-Nearest Neighbor
OTU Table	Healthy-C vs. Healthy-S	0.890	0.910	0.852	0.869	0.899	0.862
	BV-C vs. BV-S	0.882	0.873	0.838	0.844	0.870	0.836
	Healthy-C vs. BV-C	0.899	0.943	0.881	0.879	0.921	0.847
	Healthy-S vs. BV-S	0.862	0.917	0.832	0.837	0.874	0.834
	Healthy-C vs. BV-S	0.933	0.964	0.914	0.925	0.950	0.914
	Healthy-S vs. BV-C	0.926	0.955	0.910	0.912	0.937	0.899
	H-C vs. H-S vs. BV-C vs. BV-S	0.776	0.838	0.723	0.738	0.794	0.740
	Mean	0.881	0.914	0.850	0.858	0.892	0.847
	Range	0.776-0.933	0.838-0.964	0.723-0.914	0.738-0.925	0.794-0.950	0.740-0.914
10 Indices	Healthy-C vs. Healthy-S	0.995	0.998	0.994	0.989	0.997	0.979
	BV-C vs. BV-S	0.996	0.996	0.995	0.990	0.996	0.981
	Healthy-C vs. BV-C	0.717	0.697	0.588	0.588	0.692	0.678
	Healthy-S vs. BV-S	0.784	0.785	0.765	0.766	0.790	0.774
	Healthy-C vs. BV-S	1.000	0.999	1.000	0.998	0.999	0.992
	Healthy-S vs. BV-C	0.981	0.980	0.973	0.969	0.979	0.963
	H-C vs. H-S vs. BV-C vs. BV-S	0.748	0.739	0.650	0.678	0.739	0.723
	Mean	0.889	0.885	0.852	0.854	0.885	0.870
	Range	0.717-1	0.697-0.999	0.588-1	0.588-0.998	0.692-0.999	0.678-0.992
**Independent Tests with nDLBVM microbiome datasets (Samples=1076 with 251 healthy-simple and 825 healthy-complex)**							
OTU Tables	Independent evaluation with the previously learned model of Healthy-C vs. Healthy-S	0.675	0.767	0.625	0.666	0.767	0.494
10 Indices		0.850	0.836	0.854	0.846	0.836	0.842
OTU Tables	Half-by-half split of the nDLBVM data *per se* for learning (training) and performance evaluation	0.642	0.857	0.427	0.747	0.854	0.243
10 Indices		0.980	1.000	0.992	0.992	1.000	0.984
